# Feature Refine Network for Salient Object Detection

**DOI:** 10.3390/s22124490

**Published:** 2022-06-14

**Authors:** Jiejun Yang, Liejun Wang, Yongming Li

**Affiliations:** College of Information Science and Engineering, Xinjiang University, Urumqi 830046, China; yangjiejun9527@stu.xju.edu.cn (J.Y.); lym@xju.edu.cn (Y.L.)

**Keywords:** salient object detection, attention mechanism, deep learning

## Abstract

Different feature learning strategies have enhanced performance in recent deep neural network-based salient object detection. Multi-scale strategy and residual learning strategies are two types of multi-scale learning strategies. However, there are still some problems, such as the inability to effectively utilize multi-scale feature information and the lack of fine object boundaries. We propose a feature refined network (FRNet) to overcome the problems mentioned, which includes a novel feature learning strategy that combines the multi-scale and residual learning strategies to generate the final saliency prediction. We introduce the spatial and channel ‘squeeze and excitation’ blocks (scSE) at the side outputs of the backbone. It allows the network to concentrate more on saliency regions at various scales. Then, we propose the adaptive feature fusion module (AFFM), which efficiently fuses multi-scale feature information in order to predict superior saliency maps. Finally, to supervise network learning of more information on object boundaries, we propose a hybrid loss that contains four fundamental losses and combines properties of diverse losses. Comprehensive experiments demonstrate the effectiveness of the FRNet on five datasets, with competitive results when compared to other relevant approaches.

## 1. Introduction

Visual saliency can be defined as the most interesting region or object in the human vision system. The detection and segmentation of salient objects in natural scenes is often referred to as salient object detection (SOD). SOD usually serves as an important image pre-processing in many applications, including strengthening the high-resolution satellite image scene classification under unsupervised feature learning [1], unsupervised video target segmentation [2], video summarization [3], image editing and operation [4], visual tracking [5], etc. Detecting saliency objects demands a semantic comprehension of the entire image and a specific structure information of the object. Thus, saliency detection is a crucial and difficult basic problem in computer vision.

VGG [6] and ResNet [7] are examples of deep convolutional neural networks (CNNs) that have shown potential in computer vision. However, it is difficult to reconcile the problems when ResNet is applied to solve dense prediction tasks, such as medical image segmentation and scene segmentation. As the network depth becomes deeper, the spatial size of the features becomes smaller, due to the strides of multiple convolution operations. Therefore, early SOD methods are based on super-pixel [8] or based on image patches [9]. However, these frameworks do not make full use of high-level information, and spatial information cannot propagate to the last fully connected layer, which leads to the loss of global information [10]. These problems prompt the introduction of fully convolutional networks [11] into dense prediction tasks. However, there is also the difficulty of recovering the object boundary (low-level features). The difficulty prompts researchers to explore the restoration of spatial details by multi-scale architecture.

Amulet has [12] introduced a multi-scale feature aggregation strategy. The architecture utilizes the VGG-16 as the backbone to extract features and the output of its convolutional layers as features of different scales. It up-samples the features at each scale to the original scale, and then integrates all feature maps to compose the final saliency map. Due to the magnification of the up-sample operation, a lot of unnecessary information is introduced to the feature maps after unifying the scale so that the prediction ability of the network is reduced. In another way, DSS [13] introduces short connections with supervision into the Amulet-based skip-layer structures [14]. However, DSS also forces features of various scales to regress to the original input scale, then fuses different scale features to obtain the final saliency map. It faces the same problem as Amulet, due to the reduction in spatial resolution; it is difficult to accurately predict the pixel-level map after up-sampling. Although Amulet and DSS can accurately detect the salient object location, they cannot predict better for object boundaries. Subsequently, the R2Net is proposed by Feng et al. [15]. R2Net implements a residual learning strategy using the attentional residual module (ARM) [15] and dilated convolutional pyramid pooling module (DCPP) [15] to gradually adjust the prediction. In addition, R2Net conducts the reconstruction of the final saliency map in coarse-to-fine fashion. Meanwhile, the residual learning strategy obtains prior knowledge of the location of the object in the previous prediction. Then, the network obtains the final saliency map in an iterative regression process. This avoids the model from directly regressing ground-truth (GT) from different scales. However, it is undeniable that the multi-scale neural network architecture facilitates the performance of SOD. Refs. [13,16] present that in earlier convolutional layers, the features maintain high resolution and enable the reconstruction of fine boundaries. In the later convolutional layers, more semantic information of the image is retained to identify the image region, but how to effectively utilize the hierarchical network architecture is still an open question.

As for our proposed method, which reconsiders the effectiveness of multi-scale feature fusion strategy, and the recently popular attention mechanism [17], we design a new network named FRNet. FRNet employs ResNet-101 as the backbone to extract input image features and generate outputs at five scales. At first, these outputs are fed into scSE for attention processing, then fed into ARM and DCPP for residual learning. Finally, AFFM integrates features of five scales to generate the final saliency map. The hybrid loss compares predictions with GT to calculate the loss to prompt the network learning of salient features.

Our main contributions are as follows:

- We introduce an scSE module for pre-processing the outputs at five scales of the backbone. The scSE consists of channel and spatial attention. It improves the representation ability of the features at each scale and enhances the effectiveness of the residual learning strategy.
- We propose an AFFM module that enables the overall fusion of multi-scale information to compose the final saliency prediction. AFFM combines two kinds of multi-scale feature fusion strategies to improve saliency detection performance.
- A hybrid loss is proposed for supervision during five scales’ residual learning processes, and for the generation of final saliency maps. It fuses BCE, structural similarity (SSIM) and dice and intersection-over-union (IOU) to train the model in local-level and global-level.

The remainder of the work is structured as follows: the related SOD works are discussed in Section 2. In Section 3, the overall architecture of FRNet and the details of each part in FRNet are discussed. In Section 4, the experimental results of FRNet with other methods are reported. Then, the ablation experiments are conducted for each module to verify the effectiveness of the proposed modules. Finally, the paper is concluded in Section 5.

## 2. Related Work

Amulet, DSS, R3Net [18] and the R2Net method are based on the full convolutional neural network and have achieved impressive results in the SOD task. They employ holistically-nested edge detection (HED) [14] and iteratively improve network architecture and learning strategies to reconstruct different low-scale features into high-scale features. To improve the SOD, this paper combines two feature reconstruction strategies. One is residual learning through iterative refinement and another is high-resolution map reconstruction directly from all the scales. Therefore, we can also consider SOD as a reconstruction problem [19].

### 2.1. Residual Learning

The strategy for residual learning is inspired by ResNet [7], and it encodes original data, as residual tensors are more efficient than the ordinary mode. By stacking the convolutional layer and fitting the mapping function H (x), it is formulated as the residual form function H (x)=F (x)+x, *x* denotes the initial input, and also can be regarded as prior knowledge. Without using residual learning, the formula is written as $H\;\left(x\right)=F^{\prime }\left(x\right)$. We can conclude the learning strategy of DSS and Amulet, which treats the learning process on all scales as a sub-network, and each sub-network learns a mapping function to regress GT. R3Net and R2Net learn the residual mapping function to regress GT to adjust the coarse prediction at each scale. Our approach similarly employs such a strategy.

### 2.2. Multi-Scale Feature Fusion

Refs. [11,14] show that multi-scale deep features can generate better predictions [20]. Researchers have proposed many different strategies and methods for multi-scale feature fusion. Zhang et al. [21] introduce a reformulated dropout and a hybrid up-sampling module to eliminate the checkerboard effects of deconvolution operators. Luo et al. [22] present an NLDF network with a 4 × 5 grid architecture for saliency detection, in which feature maps from later layers are gradually blended with feature maps from earlier layers. Zhang et al. [23] utilize a sibling architecture to generate saliency maps by extracting feature maps from both original pictures and projection images. Wu et al. [24] propose a novel mutual learning module for improving SOD accuracy by more effectively exploiting the correlation of borders and regions. Chen et al. [25] iteratively use the side output of the backbone network as feature attention guidance to predict and refine saliency maps. For quick and accurate SOD, Wu et al. [26] offer the cascaded partial decoder (CPD). These SOD approaches make use of the multi-scale information retrieved by the backbone and outperform classic algorithms in the SOD task.

### 2.3. Attention Mechanism

It may also be considered as a dynamic selection process in computer vision that which is achieved by adaptive weighted features based on the relevance of the input data. In the saliency areas, it mimics the mechanism of the human visual system. Attention mechanisms provide performance improvements in many computer vision tasks. Some scholars have introduced the attention mechanism into SOD. Liu et al. [27] proposed PiCANet to generate attention over the context regions. Zhang et al. [28] offer a progressive attention-guiding mechanism to refine saliency maps by designing a spatial and channel attention module to acquire global information at each layer. The attention module proposed by Zeng et al. [29] computes the spatial distribution of foreground objects over image regions and aggregates the feature of all the regions. Various pure and deep self-attention networks have emerged [30,31,32], demonstrating the enormous potential of transformer models. Liu et al. [33] proposed the pure transformer for RGB and RGB-D SOD for the first time.

We utilized channel attention and spatial attention to design scSE and weight spatial attention to guide the network to learn more discriminative feature for predicting saliency map. Then, we designed the feature fusion module AFFM with multi-scale features, processing the feature at each scale after ARM, and adaptively integrated them to compose the final saliency prediction.

### 2.4. Loss Function

In addition to the discussion of the model architecture, the demand for innovating different loss functions have also become more prominent. In most of the SOD models, the binary cross-entropy (BCE) loss is often used for network training. It is formulated as follows:

(1) $$L_{BCE}=-\frac{1}{W\times H}\overset{W\times H}{\underset{i=1}{\sum }}\left(y_{i}\log x_{i}+\left(1-y_{i}\right)\log\left(1-x_{i}\right)\right),$$

where $y_{i}$ denotes ground truth pixel values, $x_{i}$ denotes prediction pixel values and $x_{i},\;y_{i}\in \left[0,\;1\right]$. 

Luo et al. [22] introduced dice loss [34] for the SOD task, and statistical supervision on the final prediction. Dice is formulated as follows:

(2) $$L_{Dice}=1-\frac{2TP}{\left(FN+TP\right)+\left(FP+TP\right)},\;$$

where TP denotes true positive, FN denotes false negative; FP denotes false positive.

Qin et al. [35] introduced *SSIM* and *IOU* loss to the traditional BCE loss. *SSIM* loss and IOU loss are formulated as follows:

(3) $$L_{SSIM}=1-\frac{\left(2\mu_{x}\mu_{y}+C_{1}\right)\left(2\sigma_{xy}+C_{2}\right)}{\left(\mu_{x}^{2}\mu_{y}^{2}+C_{1}\right)\left(\sigma_{x}^{2}+\sigma_{y}^{2}+C_{2}\right)},\;$$

(4) $$L_{IOU}=1-\frac{TP}{FN+TP+FP},$$

where $x=\left\{x_{j}:j=1,\ldots ,N^{2}\right\}$ and $y=\left\{y_{j}:j=1,\ldots ,N^{2}\right\}$ are the pixel values of two corresponding patches (size:  N×N) cropped from the predicted map and ground truth mask, respectively. In addition, $\mu_{x},\;\mu_{y},\;\sigma_{x},\;\sigma_{y}$ are the mean and standard deviation of x and y, respectively. $\sigma_{xy}$ is covariance of x,y. TP denotes true positive, FN denotes false negative; FP denotes false positive. $C_{1}=0.01^{2}\;\mathrm{and}\;C_{2}=0.03^{2}$ are used to avoid being divided by zero.

These loss functions focus on different points. BCE and SSIM focus on local supervision, calculating the gradient of each pixel based on the prediction of limited neighbored regions. Dice and IOU focus on global supervision, reducing the difference between prediction and GT. They enhance the prediction of object contour. Based on that, we propose hybrid loss, which combines these four loss functions to supervise the network learning.

## 3. Proposed Method

In Section 3.1, we show the architecture of FRNet. In Section 3.2, we show the details of the AFFM. Next, we describe the scSE module in Section 3.3. Finally, we explain the hybrid loss in Section 3.4.

### 3.1. Overview of FRNet

**Figure 1:**
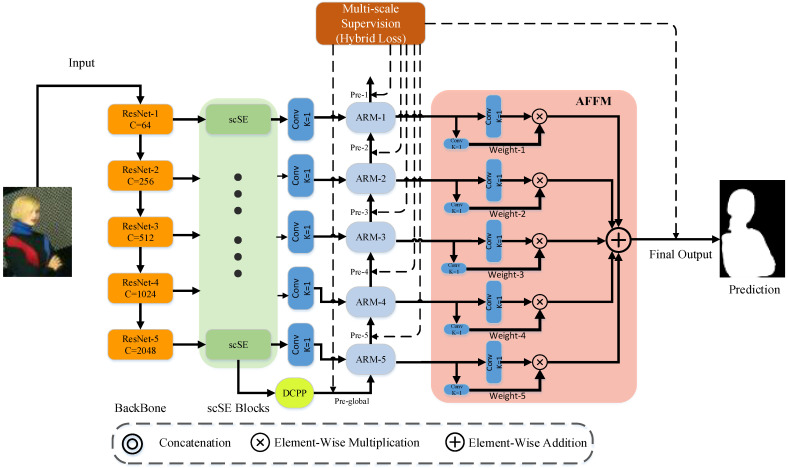
Illustration of FRNet.

**Figure 2:**
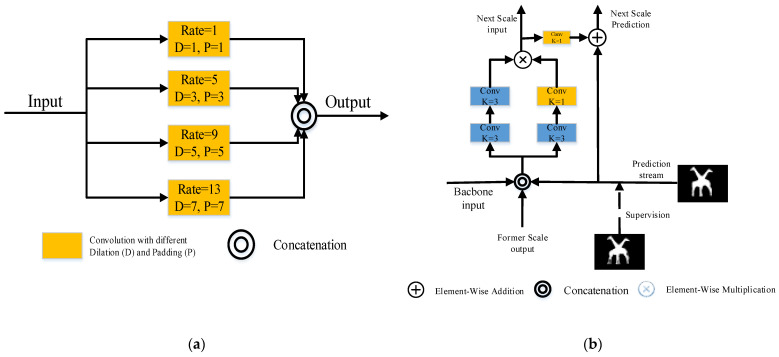
(**a**) Structure of DCPP [15] module; (**b**) details of ARM [15] module.

The feature refine network (FRNet) utilizes the full convolutional network to solve the SOD task. The entire framework is illustrated in Figure 1. The orange region is the backbone, used to extract features from the input image. The output of ResNet-5 is processed by scSE and fed into the DCPP module for multi-scale dilated convolution to coarsely predict salient objects. Details of the DCPP are shown in Figure 2a. The feature fusion strategy in skip-layer style is used in the palm blue region containing ARM blocks. The data streams are interacting with each other in every scale. Then, calculating loss with the GT at each scale, the ARM gradually adjusts the output with GT. More information about the ARM can be found in Figure 2b.

The original ResNet model is used for the image classification task, so that the spatial resolution of the feature generated by the network is mismatched to the input image resolution. To make it suitable for the SOD task, we have made the following modifications: (1) the full connection layer of the ResNet model for image classification is removed. (2) The stride in ResNet-4 is changed to 1, retaining more feature information because the feature spatial scale has not changed.

After the backbone, the outputs from each layer are processed by scSE to guide the feature maps that focus on the saliency region. These maps are fed to the ARM modules for residual learning, to adjust the prediction output gradually from a small scale to a large scale. Finally, five scale outputs after ARM processing are fed to the AFFM module for adaptive fusion. The process generates the final prediction output. The entire network is trained under the supervision of the hybrid loss.

### 3.2. Adaptive Feature Fusion Module

The main idea of AFFM is to achieve adaptively the learning of the fusion spatial weights of feature maps in various scales. As shown in Figure 1, AFFM can be divided into the following two steps: unifying scale; adaptive fusing.

Unifying scale. The saliency map of the resolution at scale S (S∈{1, 2, 3, 4, 5} for ResNet-50) is denoted as $x^{S}$. For scale S, the maps $x^{n}$ is resized from scale n (n≠S) to the same shape as $x^{S}$. Thus, AFFM can unify features on the five scales. In AFFM, it up-samples features when they are smaller than the input scale, and down-sample features when they are larger than the input scale.

Adaptive fusing. $x_{i,j}^{n\to S}$ denotes the feature tensor at the position (i, j) of the feature map, which is resized from scale n to scale S. The fusion process is formulated as follows:

(5) $$y_{i,j}^{S}=\alpha_{i,j}^{S}\cdot x_{i,j}^{1\to S}+\beta_{i,j}^{S}\cdot x_{i,j}^{2\to S}+\gamma_{i,j}^{S}\cdot x_{i,j}^{3\to S}+\delta_{i,j}^{S}\cdot x_{i,j}^{4\to S}+\varepsilon_{i,j}^{S}\cdot x_{i,j}^{5\to S},\;$$

where $y_{i,j}^{S}$ denotes the (i,j)-th tensor of the output feature maps $y^{S}$ in one of the channels. $\alpha_{i,j}^{S},\;\beta_{i,j}^{S},\;\gamma_{i,j}^{S},\;\delta_{i,j}^{S},\;\varepsilon_{i,j}^{S}$ refers to the spatial weights for the saliency map at five scales, which are adaptively calculated by the network.

We use 1 × 1 convolution layers to compute the weight scalar maps $\alpha_{i,j}^{S},\;\beta_{i,j}^{S},\;\gamma_{i,j}^{S},\;\delta_{i,j}^{S},\;\varepsilon_{i,j}^{S}$ from $x_{i,j}^{1\to S},\;x_{i,j}^{2\to S},\;x_{i,j}^{3\to S},\;x_{i,j}^{4\to S},\;x_{i,j}^{5\to S}$, respectively, so that they can be learned by standard back-propagation. By the AFFM module, the features at all scales can be adaptively aggregated. The outputs $\left\{y^{1},y^{2},y^{3},y^{4},y^{5}\right\}$ are processed by the addition operation to compose the final saliency prediction.

### 3.3. Mixed Channel and Spatial Attention

**Figure 3:**
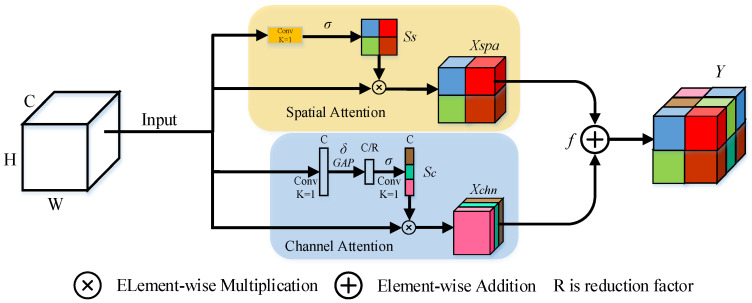
Structure of scSE.

Inspired by Roy et al. [36], we proposed the spatial and channel squeeze and excitation blocks (scSE blocks), using spatial blocks as a complement for channel SE blocks. The scSE combines two blocks directly by element-wise addition and utilizes channel attention information to improve the role of spatial attention block. Then, scSE is added to the ARM output with one channel to strengthen the representation ability of feature on each iteration. The architecture of scSE is shown in Figure 3.

The channel attention is formulated as follows:

(6) $$s_{c}=\sigma \left(W_{2}\delta \left(W_{1}GAP\left(X\right)\right)\right),\;$$

(7) $$X_{chn}=s_{c}X,$$

where *W* denotes the filter kernel weight and *GAP* denotes the global average pooling. *GAP* uses the nn.AdaptiveAvgPool2d method for global average pooling operations.

Spatial attention is formulated as follows:

(8) $$s_{s}=\sigma \left(Conv^{1\times 1}\left(X\right)\right),\;$$

(9) $$X_{spa}=s_{s}X,$$

where σ denotes sigmoid activation, and δ denotes ReLU activation.

scSE is formulated as follows:

(10) $$Y=f\left(X_{spa},X_{chn}\right),$$

where f denotes the fusion function, which we set it to element-wise addition operation.

### 3.4. The Hybrid Loss

The common loss functions in the SOD task are divided into two categories. For local supervision, such as *BCE* and *SSIM*, these are unable to supervise the network to learn fine object boundaries. For global supervision, dice and *IOU* can detect finer object boundaries. Naturally, we consider the model to achieve better performance when it is training with hybrid loss. The hybrid loss is defined as follows:

(11) $$L_{FL}=\alpha \left(L_{BCE}+L_{SSIM}\right)+\beta \left(L_{Dice}+L_{IOU}\right),$$

where α,β are the proportion parameter; in this paper, we set it to 0.4 and 0.6 and allowed the model to focus more on the prediction of the object boundary.

These loss functions complement each other. *BCE* and *SSIM* supervise the network as a local loss to prompt the network learning of the approximate location of the salient object. Dice and *IOU* supervise the network as a global loss to prompt the network learning of the fine boundary of the salient object. FRNet employs the hybrid loss on the predicted output branch of the ARM blocks at all scales, as well as the final output after AFFM processing, as shown in Figure 1. On the one hand, multi-scale supervision prompts the network to learn the salient features more efficiently. On the other hand, it also improves the model prediction accuracy. Each ARM module predicts a saliency map that can be expressed as $M^{\left(i\right)},$ where i∈{1,2,3,4,5}. They correspond to the prediction output after ARM block processing on the five scales, and each one calculates the loss. Equation (11) is further expressed as follows:

(12) $$L_{HL}=\overset{5}{\underset{i=1}{\sum }}(\alpha \left(L_{BCE}^{i}\left(M^{i}\right)+L_{SSIM}^{i}\left(M^{i}\right)\right)+\beta \left(L_{Dice}^{i}\left(M^{i}\right)+L_{IOU}^{i}\left(M^{i}\right)\right)),$$

More experiments about ablation of the hybrid loss can be found in Section 4.5.

## 4. Experiments

To validate the FRNet network, we conduct the comparison experiment on five public datasets (including ECSSD [37], PASCAL-S [38], DUT-OMRON [39], HKU-IS [40], DUTS [41]). DUTS is used for the training of the model. The other datasets are used for the evaluation of the model. We choose precision–recall curves (PR), mean absolute error (MAE), S-measure (Sm) and maximum F-measure (Max-F) as the evaluation metrics for the experiments. The baseline is selected as R2Net.

### 4.1. Implementation Details

#### 4.1.1. Data Augmentation

Inspired by Liu et al. [42], the images are randomly vertical- and horizontal-flipped, and randomly rotated to overcome the over-fitting of the model. Subsequently, brightness, contrast, saturation and hue of images are randomly changed to enhance the generalization ability of the model.

#### 4.1.2. Parameter Setting

The model code is implemented on public platform PyTorch, with two Tesla V100 GPUs (with 16 GB memory) for the experiments. At first, each image is resized to 384 × 384 and performed normalization. During the training, epoch is set to 40, batch-size is 16. The momentum is set to 0.9 and weight decay is 0.0005. Then, we set the base learning rate (lr) to 0.0005 and the learning rate multiplies 0.1 every 10 epochs. FRNet employs the Adam optimizer and hybrid loss to optimize the network. The “Kaiming” method initializes the convolutional layers of ResNet. 

### 4.2. Datasets

We evaluate the saliency detection performance of FRNet on five benchmark datasets.

ECSSD [37] contains 1000 natural images with different sizes and contains multiple objects. Some images are derived from the challenging Berkeley-300 dataset [43].

PASCAL-S [38] has 850 images selected from the validation set of PASCAL VOC2010 [44] used for the segmentation task.

DUT-OMRON [39] contains 5172 images that are carefully annotated by 5 testers, and hand-picked from over 140,000 natural images, each containing one or more saliency objects, along with associated intricate backgrounds.

HKU-IS [40] includes 4447 images, with high-quality pixel-level labels. Images are selected carefully, containing multiple disconnected objects or the object itself overlaps the background.

DUTS [41] allocates 10,533 images for training, and 5019 images for validation. The training images are collected from ImageNet DET training set [45]. The images of validation are collected from ImageNet DET test set and the SUN dataset [46] with pixel-level annotation.

### 4.3. Evaluation Metrics

#### 4.3.1. Precision–Recall (PR) Curves

At first, the saliency map *S* is converted into a binary mask *M*. Precision and recall are calculated by *G* and *M*. *G* denotes ground-truth. PR is formulated as follows:

(13) $$Precision=\frac{\left|M\cap G\right|}{\left|M\right|},\;Recall=\frac{\left|M\cap G\right|}{\left|G\right|},$$

#### 4.3.2. Maximum F-Measure (Max-F)

Precision and recall cannot fully evaluate the quality of the prediction map, in other words, the high precision and recall are both required. So, F-measure is proposed as the weighted harmonic mean of precision and recall with a non-negative weight $\beta^{2}$. The formula of Max-F as follows:

(14) $$F_{\beta }=\frac{\left(1+\beta^{2}\right)Precision\times Recall}{\beta^{2}Precision+Recall},$$

When the max-F value is larger, the model performance is better.

#### 4.3.3. Structure-Measure (Sm)

Sm evaluates the structural details of the prediction map, and combines region-aware and object-aware structural similarity evaluation to obtain the final formulation of structure-measure, which is as follows:

(15) $$S=\alpha \times S_{o}+\left(1-\alpha \right)\times S_{r}$$

where $S_{r}$ denotes the region-aware structural similarity measure; $S_{o}$ denotes the object-aware structural similarity measure. For more details, readers may refer to Fan et al. [47].

A larger value of Sm indicates the better performance of the model.

#### 4.3.4. Mean Absolute Error (MAE)

The above metrics have not taken the prediction of the non-salient pixels into account, that is, the pixels correctly labeled as non-salient. For this purpose, the MAE is calculated by saliency map *S* and binary GT. *S* and *M* are pre-normalized to the range of [0,1]. The formulation of MAE is as follows:

(16) $$\mathrm{MAE}=\frac{1}{W\times H}\overset{W}{\underset{x=1}{\sum }}\overset{H}{\underset{y=1}{\sum }}\|S\left(x,y\right)-G\left(x,y)\right\|,$$

A smaller value of MAE indicates the better performance of model.

### 4.4. Comparisons with Other Advanced Methods

FRNet compares with Amulet [12], BASNet [35], PiCANet [27], CPD [26], DSS [13], F3Net [48], GCPA [49], ITSD [50], MINet [51], NLDF [22], PoolNet [52], MLMS [24], MPI [53], RCSB [54] and R2Net [15], respectively. The number in parentheses represents the year of publication of the model. The evaluation data of the models used for comparison are implemented by ourselves, or computed by their original implementation for fair comparison. Detailed information is debriefed in Table 1. Compared to other models, FRNet achieves competitive results on five public datasets.

#### 4.4.1. Visual Comparison

**Figure 4:**
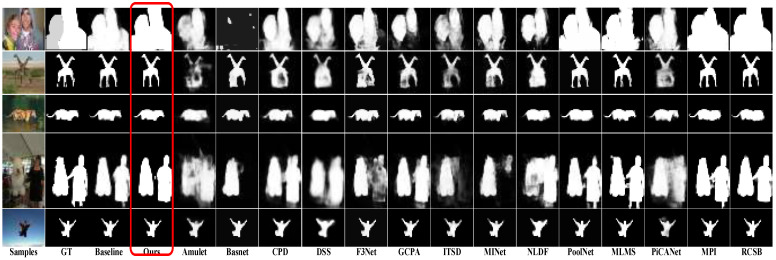
These saliency maps are generated by Amulet, BasNet, PiCANet, CPD, DSS, F3Net, GCPA, ITSD, MINet, NLDF, PoolNet, MLMS, MPI, RCSB and R2Net, respectively. FRNet generates predictions that are the best, especially for the object boundary details.

As shown in Figure 4, the results of FRNet against the other methods can be observed. FRNet accurately predicts the salient objects of all images. For objects that are easy to misjudge and connected with other objects, FRNet performs better than all other methods. These results indicate that our method is more robust. In the first row, Amulet and Basnet are unable to predict the saliency objects of the test image. For the overlapping and connected saliency objects, such as row 2 and 4, most of the methods cannot predict effectively. FRNet generates the finest object boundaries compared to other models. It also proves that scSE, loss, and AFFM all provide improvement to the FRNet. More experiment information on the three components can be found in Section 4.5.

#### 4.4.2. Evaluation of Saliency Map

**Figure 5:**
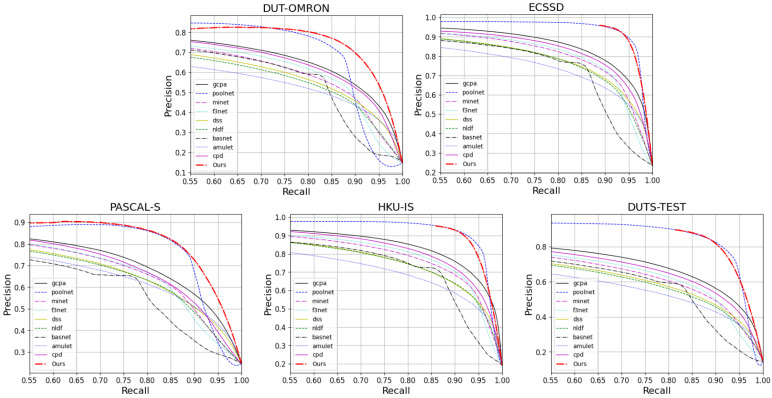
The PR curves of FRNet and the other nine advanced models on five datasets.

**Figure 6:**
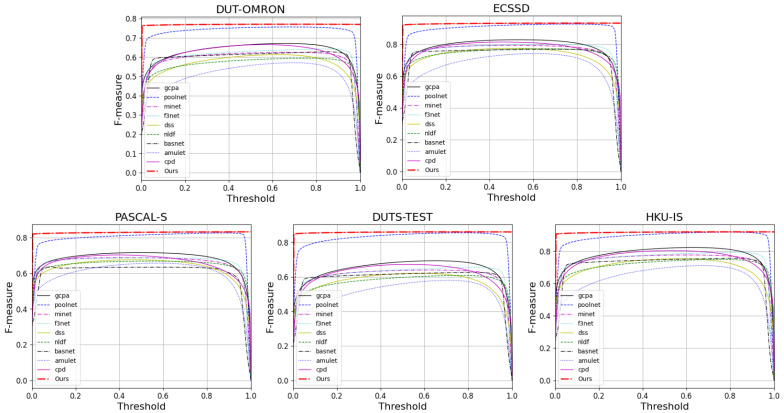
The F-measure curves of FRNet and the other nine advanced methods on five datasets.

We evaluate the prediction maps on PR Curves and F-measures. Figure 5 and Figure 6 visualize the results of these. It can be observed that FRNet outperforms most of the models. In Figure 5, FRNet maintains a higher precision value at a higher recall value. In Figure 6, FRNet also maintains a high F-measure value at high thresholds. This indicates that FRNet generates saliency maps closer to GT, with higher confidence in the saliency target region. This allows FRNet to predict the location of salient objects and to segment it finely.

### 4.5. Ablation Analysis

We carry out experiments on five public datasets to analyze the validity of hybrid loss, AFFM and scSE.

#### 4.5.1. The Effectiveness of the Adaptive Feature Fusion Module

**Figure 7:**
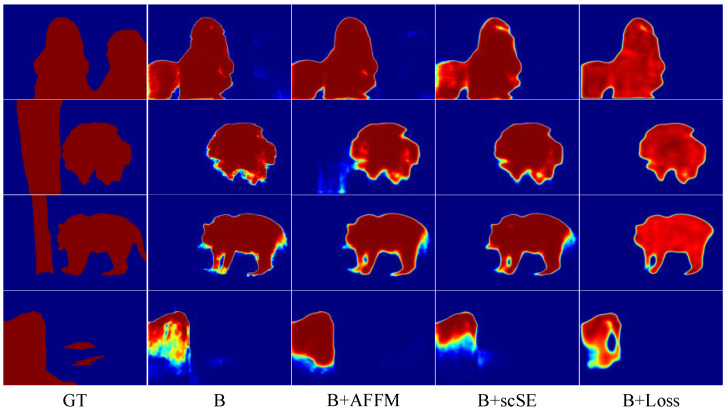
The comparison of different combinations with the backbone.

To demonstrate the effectiveness of AFFM, we design a set of experiments on AFFM by only adding AFFM modules to the baseline and then comparing results with the baseline. The first and second row result in Table 2, showing the comparison results. According to the results, it shows that the MAE measure is greatly improved compared with the baseline, but the other two metrics decreased in different degrees on ECSSD, DUT-OMRON, and HKU-IS. The Sm improved on PASCAL-S and DUT-TEST. The results demonstrate the validity of the AFFM module. As shown in Figure 7, in comparison with other methods, it shows that the backbone with the AFFM module has fewer misjudgments of salient pixels and finer boundaries.

As shown in Table 2, the results of the first four rows show that different components play different roles in the baseline. At the same time, due to the differences among the five datasets, different components have different effects on each dataset. Then, when three components are combined together, it can be constructed as a complete model architecture. In addition, the multi-scale feature fusion strategy and the residual learning strategy can be effectively combined to obtain better performance. This is reflected in the last four rows of Table 2.

#### 4.5.2. The Effectiveness of Hybrid Loss

Firstly, we conduct ablation experiments for different combinations of loss functions. As shown in Table 3, the three combinations of BCE+SSIM, Dice+IOU and BCE+Dice play a certain role in improving the MAE metric of the model. However, the other two metrics cannot be effectively improved. Finally, we combine three combinations to form the hybrid loss and obtain the best model performance.

Secondly, we conduct ablation experiment on the hybrid loss, as shown in Table 4. On the other hand, we perform experiments on the parameters of α,β in the hybrid loss, as shown in Table 4. It shows that the combination of loss with the different proportions of α,β has different effects on the model. FRNet does not perform well when the α or β is too large or too small. The characteristics of the loss functions and the regulatory focus are not coordinated. By comparing the results in the second and fourth rows, we need to assign a higher proportion to β appropriately. If in this case, we will observe that the model achieves the best performance. This kind of hyper-parameter setting is also adopted as the best setting in experiments. As shown in Figure 7, the hybrid loss effectively improves the model to detect salient objects, highlights the boundary of salient objects, and enhances the segmentation performance of the model.

#### 4.5.3. The Effectiveness of scSE

To investigate the role of scSE in the model, we carry out the ablation experiments of scSE. The results are shown in rows 1 and 4 of Table 2. The scSE improves most of the metrics on the five datasets. The results also demonstrate the effectiveness of scSE in the FRNet. As shown in Figure 7, the scSE module facilitates the model to predict salient regions, and suppresses the attention of the model on non-salient regions. Comparing the results of rows 2, 3, 5 and 7 in Table 2, the scSE module combined with other modules can be more effective in improving the detection performance of the model.

## 5. Conclusions and Discussion

A novel FRNet for SOD is proposed in this paper. To construct the final prediction, FRNet gradually optimizes the prediction results from a low to a high scale and obtains information from optimized features at all scales, until the results match GT as closely as possible. We propose a hybrid loss supervised method to obtain the object boundary information to solve the problem of the coarse object boundary. In the ablation experiments, we demonstrate the effectiveness of the hybrid loss. The extensive experimental results indicate the efficiency of the proposed strategy and FRNet. However, there are still possibilities to improve our network architecture, and we will continue to investigate the potential of various network architectures in the field of SOD, such as transformers.

## Figures and Tables

**Table 1:** The MAE, Max-F, Sm of 15 methods on 5 datasets. The top two results are in red and blue.

Method	ECSSD	PASCAL-S	DUT-OMRON	DUT-TEST	HKU-IS
	MAE/Max-F/Sm	MAE/Max-F/Sm	MAE/Max-F/Sm	MAE/Max-F/Sm	MAE/Max-F/Sm
Amulet (17)	0.0690/0.9150/0.8840	0.1000/0.8280/0.8180	0.0980/0.7430/0.7810	0.0840/0.7780/0.7960	0.0510/0.8970/0.8860
NLDF (17)	0.0630/0.9030/0.8750	0.0980/0.8220/0.8030	0.0790/0.7530/0.7500	0.0650/0.8160/0.8050	0.0480/0.9020/0.8780
PiCANet (18)	0.0460/0.9310/0.9140	0.0770/0.8570/0.8500	0.0640/0.8200/0.8080	0.0500/0.8630/0.8500	0.0440/0.9200/0.9050
DSS (19)	0.0520/0.9160/0.8820	0.0960/0.8360/0.7970	0.0740/0.7600/0.7650	0.0650/0.8130/0.8120	0.0500/0.9000/0.8780
MLMS (19)	0.0450/0.9280/0.9110	0.0740/0.8550/0.8440	0.0640/0.7740/0.8090	0.0480/0.8520/0.8510	0.0390/0.9210/0.9070
CPD (19)	0.0370/0.9390/0.9180	0.0710/0.8610/0.8480	0.0560/0.7970/0.8250	0.0430/0.8650/0.8580	0.0340/0.9250/0.9050
PoolNet (19)	0.0390/0.9440/0.9210	0.0750/0.8650/0.8320	0.0560/0.8080/0.8360	0.0400/0.8800/0.8710	0.0330/0.9320/0.9170
BASNet (19)	0.0370/0.9420/0.9160	0.0760/0.8540/0.8380	0.0560/0.8050/0.8360	0.0470/0.8600/0.8530	0.0320/0.9280/0.9090
GCPA (20)	0.0350/0.9431/0.9264	0.0712/0.8632/0.8561	0.0560/0.8223/0.8391	0.0364/0.8781/0.8715	0.0322/0.9284/0.9144
ITSD (20)	0.0346/0.9393/0.9249	0.0712/0.8354/0.8617	0.0608/0.7916/0.8404	0.0408/0.8669/0.8851	0.0307/0.9257/0.9169
MINet (20)	0.0330/0.9440/0.9030	0.0780/0.8610/0.8480	0.0560/0.8100/0.8220	0.0370/0.8840/0.8750	0.0330/0.9310/0.9140
F3Net (20)	0.0330/0.9250/0.9240	0.0680/0.8400/0.8550	0.0530/0.7660/0.8380	0.0350/0.8400/0.8751	0.0280/0.9100/0.9210
MPI (21)	0.0318/0.9415/0.9252	0.0690/0.8381/0.8607	0.0560/0.7798/0.8336	0.0348/0.8749/0.8891	0.0267/0.9317/0.9221
RCSB (22)	0.0335/0.9355/0.9218	0.0684/0.8311/0.8597	0.0490/0.7729/0.8352	0.0329/0.8667/0.8810	0.0268/0.9334/0.9188
Baseline (R2Net)	0.0440/0.9350/0.9150	0.0750/0.8280/0.8470	0.0610/0.7715/0.8240	0.0500/0.8582/0.8610	0.0390/0.9210/0.9030
Ours	0.0326/0.9457/0.9286	0.0671/0.8675/0.8590	0.0491/0.8336/0.8473	0.0339/0.8812/0.8761	0.0273/0.9323/0.9198

**Table 2:** Comparison of different combinations of three components. B denotes the baseline. AFFM denotes adaptive feature fusion module. scSE denotes channel-spatial attention. Loss denotes hybrid loss. The best results are in bold.

Method	ECSSD	PASCAL-S	DUT-OMRON	DUT-TEST	HKU-IS
	MAE/Max-F/Sm	MAE/Max-F/Sm	MAE/Max-F/Sm	MAE/Max-F/Sm	MAE/Max-F/Sm
B	0.0440/0.9350/0.9150	0.0750/0.8280/0.8470	0.0610/0.7715/0.8240	0.0500/0.8582/0.8610	0.0390/0.9210/0.9030
B+AFFM	0.0363/0.9245/0.9093	0.0706/0.8269/0.8506	0.0498/0.7570/0.8161	0.0344/0.8526/0.8695	0.0296/0.9136/0.9026
B+Loss	0.0354/0.9295/0.9110	0.0690/0.8319/0.8542	0.0502/0.7643/0.8192	0.0351/0.8539/0.8666	0.0294/0.9168/0.9020
B+scSE	0.0356/0.9285/0.9103	0.0701/0.8292/0.8503	0.0512/0.7655/0.8210	0.0347/0.8583/0.8701	0.0300/0.9151/0.9015
B+scSE+Loss	0.0327/0.9327/0.9164	0.0697/0.8325/0.8528	0.0506/0.7710/0.8252	0.0345/0.8600/0.8738	0.0285/0.9200/0.9068
B+Loss+AFFM	0.0366/0.9286/0.9063	0.0691/0.8303/0.8509	0.0521/0.7622/0.8128	0.0374/0.8455/0.8564	0.0318/0.9107/0.8935
B+scSE+AFFM	0.0328/0.9357/0.9174	0.0681/0.8325/0.8553	0.0506/0.7663/0.8221	0.0358/0.8545/0.8695	0.0288/0.9191/0.9064
B+scSE+Loss+AFFM	**0.0326/0.9367/0.9180**	**0.0671/0.8370/0.8590**	**0.0491/0.7724/0.8279**	**0.0339/0.8624/0.8761**	**0.0273/0.9223/0.9098**

**Table 3:** Comparison of different combinations of loss functions. The summation of loss functions is without extra parameters. The bold number indicate the best result.

Method	DUT-OMRON	DUT-TEST	HKU-IS
	MAE/Max-F/Sm	MAE/Max-F/Sm	MAE/Max-F/Sm
Baseline (BCE)	0.0610/**0.7755**/0.8240	0.0500/**0.8582**/0.8610	0.0390/**0.9210**/**0.9030**
Dice+IOU	0.0569/0.7554/0.8159	0.0418/0.8481/0.8518	0.0355/0.9046/0.8980
BCE+Dice	0.0525/0.7561/0.8156	**0.0359**/0.8540/**0.8682**	**0.0308**/0.9145/0.9027
BCE+SSIM	**0.0513**/0.7581/**0.8160**	0.0373/0.8436/0.8554	0.0330/0.9126/0.8913
BCE+SSIM+Dice+IOU	0.0532/0.7524/0.8079	0.0366/0.8385/0.8610	0.0334/0.9075/0.8991

**Table 4:** Ablation analysis of hybrid loss hyper-parameters. α denotes ration of (BCE+SSIM), β denotes ration of (Dice+IOU). The best results are in bold.

Method	DUT-OMRON	DUT-TEST	HKU-IS
	MAE/Max-F/Sm	MAE/Max-F/Sm	MAE/Max-F/Sm
Baseline (BCE)	0.0610/**0.7755**/0.8240	0.0500/0.8582/0.8610	0.0390/0.9210/0.9030
α=0.1, β=0.9	0.0523/0.7520/0.8082	0.0380/0.8432/0.8518	0.0337/0.9080/0.8908
α=0.6, β=0.4	0.0541/0.7529/0.8084	0.0408/0.8322/0.8470	0.0346/0.9081/0.8887
α=0.9, β=0.1	0.0538/0.7475/0.8015	0.0399/0.8344/0.8430	0.0360/0.9048/0.8820
α=0.4,β=0.6	**0.0491**/0.7724/**0.8279**	**0.0339/0.8624/0.8761**	**0.0273/0.9223/0.9098**

## Data Availability

Publicly available datasets were analyzed in this study. The training dataset DUTS can be obtained at: http://saliencydetection.net/duts/ (accessed on 15 January 2022). The testing datasets of DUT-OMRON, ECSSD, PASCAL-S, HKU-IS are available at: http://saliencydetection.net/dut-omron/, https://www.cse.cuhk.edu.hk/leojia/projects/hsaliency/dataset.html, https://cbs.ic.gatech.edu/salobj/ and https://sites.google.com/site/ligb86/mdfsaliency/ (Above URLs were accessed on 15 March 2022).
